# Fondaparinux: Should It Be Studied in Patients with COVID-19 Disease?

**DOI:** 10.1055/s-0040-1719232

**Published:** 2020-10-16

**Authors:** Francesco Marongiu, Doris Barcellona

**Affiliations:** 1 Department of Medical Sciences and Public Health, University of Cagliari, Cagliari, Italy; 2 SHRO, Temple University, Philadelphia, Pennsylvania, United States

We have read with interest the review article by Bikdeli et al recently published in *Thrombosis and Haemostasis* on the priority in planning studies on the different anticoagulants in coronavirus disease 2019 (COVID-19) infection. 1 The authors describe the characteristics of both the anticoagulants available today and in the future. Antiplatelets drugs have been also considered.

Surprisingly, fondaparinux (FPX) has reached a lower priority research mean (4.89) than unfractionated heparin (UFH) and low-molecular weight heparin (LMWH) at intermediate (7.82) or therapeutic (7.53) dosage for hospitalized ward and intensive care unit patients.

We believe that properties of these drugs have been overlooked by the scientific panel of the Global COVID-19 Thrombosis Collaborative Group.

## Guidelines on the Use of Fondaparinux in Thromboembolic Complications

FPX is a synthetic molecule recommended as LMWH in the guidelines for prophylaxis 2 and treatment of deep vein thrombosis and pulmonary embolism. 3 4 In addition, its use in the treatment of superficial venous thrombosis is recommended for approximately 45 days. 5

Guidelines propose different advises on the use of FPX in COVID-19 patients. The Consensus Statement by Zhai et al 6 and the Subcommittee of the International Society on Thrombosis and Haemostasis 7 suggest only LMWH for prophylaxis and treatment of venous thromboembolism in patients with mild or moderate COVID-19 infection. In the latter FPX use has been considered with some concern because of its long half-life and lack of an antidote. On the contrary, in CHEST guidelines 8 LMWH or FPX is suggested either for thromboprophylaxis or treatment in COVID-19 patients with deep vein thrombosis or hemodynamically stable pulmonary embolism.

## Heparin-Induced Thrombocytopenia in COVID-19

Heparin-induced thrombocytopenia (HIT) has been recently described in patients with COVID-19 treated with UFH or LMWH. The cumulative incidence of detectable HIT antibodies was 12% at 25 days in 88 patients who received at least 5 days of UFH 9 while elevated level of antiheparin-PF4 antibodies was observed in most patients treated with LMWH suggesting that HIT can contribute to the fatal outcome in COVID-19 patients in critical condition. 10

Only another case series of three patients 11 was published suggesting that the diagnosis of HIT in COVID-19 patients could be underestimated. Clinicians must be aware of the possibility of HIT in patients with COVID-19 treated with UFH or LMWH since early diagnosis is crucial to properly treat this condition while a delayed recognition may contribute to poor outcomes.

## Treatment Option with Fondaparinux in HIT

FPX has several advantages over UFH or LMWH: bioavaibility (100 vs. 30% and 90% respectively), no binding to protein, endothelial cells, and macrophages. 12 The possibility of being given only once a day subcutaneously and its long half-life (17–21 hours) make it simple and comfortable in everyday use. Its limitations are represented by the lack of an antidote although andexanet is potentially a reversal agent 13 and by a severe kidney failure (GFR <30 mL/min) that can induce accumulation of the drug. 14 On the other hand, LMWH have these limitations too.

FPX does not induce antiheparin-PF4 antibodies. Even if FPX is off label for the treatment of HIT, it seems an appropriate anticoagulant to use in this clinical setting as we describe in a case of severe HIT with arterial and venous thrombosis 15 and as recently stated by Warkentin. 16 Moreover, a recent expert opinion of the Section on Pulmonary Circulation of the Polish Cardiac Society 17 suggests to consider the use of FPX in patients with COVID-19 infection.

## Nonanticoagulant Actions of Fondaparinux

What do we know about its possible antiviral and anti-inflammatory properties?

As reported by the authors, in vitro studies showed that heparin is able to significantly limit Zika virus-induced cell death of human neural progenitor cells. 18 Although the mechanism is unknown, heparin and heparin sulfate show a strong binding to adeno-associated virus (AAV2) but this effect disappeared when delsulfated polysaccharides were challenged indicating that the interaction with AAV2 is due to the sulfonated regions of the heparin molecules. 19 The same result was obtained employing FPX whose structure differs from that of both heparin and heparin sulfate for a terminal *o* -methyl group but it is quite similar to the sequences prevailing in heparin and heparan sulfate to which several AAVs bind during entry. 20 In other words, FPX is a sulfated molecule whose antiviral properties should be studied in a more extensive way in the future.

As far as FPX's anti-inflammatory properties are concerned, a recent study 21 has showed that this drug is effective in increasing the survival of sepsis-induced baboons by *Escherichia coli* . Moreover, the authors demonstrated that FPX treatment greatly reduces production of inflammatory cytokines and chemokines such as tumor necrosis factor, interleukin-β (IL‐β), IL‐6, IL‐8, monocyte chemoattractant protein‐1, and granulocyte‐macrophage colony‐stimulating factor.

It is worth noting that the same profound inflammatory response plays an important role in the COVID-19-infected patients. 22 In both conditions, i.e., viral and bacterial infections, the role of procoagulant monocytes, upon stimulation by cytokines and chemokines, is crucial since they can activate blood coagulation by exposing tissue factor on their surface. 23 These mechanisms are to be expected since the innate immunity and hemostasis are ancestral defensive mechanisms aimed at trapping virus and bacteria in a fibrin network. 24 As a consequence, in COVID-19 infection disease, pulmonary thrombosis, 25 venous thromboembolism, and disseminated intravascular coagulation may occur 26 thus significantly worsening the disease.

## Conclusion

All these remarks indicate that anticoagulation is important in such a disease and that FPX may be a drug of choice, compared with LMWH, in the COVID-19 infection and deserves, in our opinion, a better place in the priority in a future research programs on this topic.

A prospective observational cohort study would be appropriate using an intermediate or therapeutic dose of FPX. Prospective cohort studies may show both the course and long-term complications of the disease. They would also allow observing whether anticoagulation is associated with a decrease in mortality and, through regression models, these studies would allow to investigate on predictors of mortality as well as drug toxicity.
